# Lateral Sinus Floor Elevation Performed with Trapezoidal and Modified Triangular Flap Designs: A Randomized Pilot Study of Post-Operative Pain Using Thermal Infrared Imaging

**DOI:** 10.3390/ijerph15061277

**Published:** 2018-06-16

**Authors:** Antonio Scarano, Felice Lorusso, Merla Arcangelo, Camillo D’Arcangelo, Renato Celletti, Pablo Santos de Oliveira

**Affiliations:** 1 Department of Medical, Oral and Biotechnological Sciences and CeSi-Met, ‘G. D’Annunzio’ University of Chieti-Pescara, Via dei Vestini 31, 66100 Chieti, Italy; camillo.darcangelo@unich.it (C.D.); rcelletti2016@gmail.com (R.C.); 2 Department of Medical, Oral and Biotechnological Sciences University of Chieti-Pescara, Via dei Vestini 31, 66100 Chieti, Italy; drlorussofelice@gmail.com; 3 Department of Neuroscience, Imaging and Clinical Sciences, ‘G. D’Annunzio’ University of Chieti-Pescara, Via dei Vestini 31, 66100 Chieti, Italy; arcangelo.merla@unich.it; 4 Department of Oral Implantology, Dental Research Division, College Ingà, UNINGÁ, 29312 Cachoeiro de Itapemirim, Espirito Santo, Brazil; psoliveiraodonto@yahoo.com.br

**Keywords:** trapezoidal flap, modified triangular flap, sinus augmentation, VAS, VRS, thermal infrared imaging

## Abstract

*Purpose:* Post-operative pain and swelling are frequently observed after sinus lift procedures. The aim of the present study was the clinical evaluation of swelling and pain of two different sinus flap lift techniques using a visual analogue scale (VAS), verbal rating scale (VRS), and infrared thermal imaging (i.e., thermography). *Materials Methods*: A randomized controlled trial was conducted with 15 patients (30 sinuses in total) randomly allocated into two groups. For the sinuses of Group I a trapezoidal flap was used, while for Group II a modified triangular flap without anterior release was utilized. Postoperative pain was scored by means of a 100-mm VAS ranging from 0 (no pain) to 100 (worst pain imaginable), and was recorded at 2, 4, 6 and 14 days after surgery. Swelling was recorded by a verbal rating scale (VRS) and was classified into four categories: a score of 1 referred the absence of swelling, patients with intra-oral swelling in the surgical zone scored 2, any extra-oral swelling in the surgical zone scored 3, and intense swelling exhibited by extra-oral swelling extending beyond the surgical zone scored 4. The facial temperature was recorded before and after sinus augmentation, and at 2, 4, 6, and 14 days post-surgery to check the course of healing. *Results*: In Group I pain intensity was recorded at 2 days after surgery with a mean score of 38.67 ± 6.4 mm. Swelling was greater at 2 and 4 days, and was absent at day 6. The facial temperature difference before and after the procedure was 4.737 °C ± 0.37. In Group II the pain score were lower than in Group I (*p* < 0.05). The score for swelling was 2 on the first and second days, and was reduced on day 4. After the second day the difference in temperature was significantly reduced as compared to the day of surgery (0.77 °C); at 2 and 4 days no difference was registered. *Conclusions*: The results of this clinical study show the significant effectiveness of the modified triangular flap in the sinus lift procedure for reducing pain and swelling.

## 1. Introduction

Post-operative pain and swelling are frequently observed after sinus lift procedures. Orofacial pain management is a challenging topic for the dental-medical profession [1].

The placement of dental implants in the distal edentulous maxillary area is often a problem in case of the severely resorbed maxilla and presents a complex challenge to the oral and maxillofacial surgeon [2,3,4,5]. Inadequate bone height in the lateral part of the maxilla forms a contra-indication for implant surgery; due to the significant resorption in the posterior maxilla following tooth extraction and the pneumatization of the maxillary sinus, there is not enough bone volume to ensure primary stability of dental implants [6,7,8,9]. Different anatomical factors influence new bone formation; in fact the healing and mineralization processes after sinus lifting are negatively correlated with the width of the bucco-palatal sinus [10,11], with the width of the bony window during lateral sinus augmentation [12,13], and with reduced crestal height [14,15].

Meanwhile, there is a positive correlation with the number of sinus walls in contact with the grafting material in cases of large sinus cavities because new bone formation starts from the residual crest and continues in a centripetal direction [16].

The transcrestal approach as a treatment option may be preferred in case of narrow sinus cavities [10].

The goals of sinus augmentation procedures are: formation of vital bone, implant integration in newly-formed bone, and long-term implant survival after functional load [17,18,19,20,21,22]. 

Since there are a large number of bone substitutes available, the properties of these materials, related to biocompatibility and function in comparison to the gold standard, must be considered by surgeons in making recommendations and assisting in the patient’s treatment-planning process. Various grafting materials have been used for sinus augmentation: autologous bone, mineralized and demineralized freeze-dried allografts, coralline calcium carbonate, bioglass, polylactide-polyglicolide materials, synthetic polymers, calcium sulfate, anorganic bovine bone, and hydroxyapatite [7,23,24,25,26,27]. The sinus augmentation procedure in the lateral sinus floor elevation causes discomfort in the first three days after surgery, although this can vary from person to person [28,29,30]. The patients present with swelling or bruising. Typically, the worst swelling occurs on the third day, and then gradually decreases. It is difficult to evaluate pain and swelling in sinus surgery. To overcome these difficulties, we used infrared thermography. The purpose of the present study was a clinical evaluation of swelling and pain with two different types of flap for sinus lift, assessed through infrared thermal imaging (i.e., thermography) and the visual analogue scale.

## 2. Materials and Methods

### 2.1. Inclusion and Exclusion Criteria

The protocol for this study was designed in accordance with the Helsinki Declaration (revised version of Tokyo at 2004) and Good Clinical Practice Guidelines. It was approved by the Inter Institutional Ethics Committee of Faculdade Ingá, UNINGÁ, PR, Brazil, No. 72105917.5.0000.5220.

Fifteen healthy patients with non-contributory past medical history (9 women and 6 men, all non-smokers, mean age 53 years, range 45–61 years) were included in this randomized pilot study. A total of 30 sinuses were randomly allotted into two groups (15 sinuses each). All patients (candidates for augmentation in the posterior maxilla) were scheduled to receive fixed prosthesis or crown restorations. All patients signed a written informed consent form. All patients were treated at the Department of Oral Implantology, Center for Advanced Studies, Dental Research Division, UNINGÁ—Cachoeiro de Itapemirim, Brazil. At the initial visit, all subjects underwent a clinical and occlusal examination. Panoramic radiographs were evaluated as well.

The inclusion criteria were: edentulous or partly edentulous patients with a unilateral or bilateral loss of teeth in the maxillary premolar or molar areas, with severe alveolar atrophy and a residual alveolar ridge height of between 2 and 4 mm. The exclusion criteria were: severe illness, head and neck radiation therapy, chemotherapy, uncontrolled diabetes, uncontrolled periodontal disease, and smoking. Exclusion criteria also included facial or neck inflammatory skin diseases, carotid sinus hyperesthesia, hyperthyroidism, and patients who unilaterally declined undergoing post-operative treatment.

Of these patients, one patient was totally edentulous in the maxilla, and 19 others were partially edentulous in the posterior maxilla. After a thorough oral and physical examination, patients were scheduled for bone reconstruction procedures, including sinus augmentation and implant insertion. Preoperatively, they were extensively informed concerning the surgical procedures and they were asked for their full cooperation during treatment. A three-dimensional radiographic examination (CBCT) before sinus augmentation was performed on all patients to identify clinically relevant and radiographically evident pathologies such as mucosal thickening, allergic sinusitis, odontogenic sinusitis, mucus-retaining cysts partial to complete sinus obliteration, oroantral communications, antroliths, mucoceles, and mucopyoceles. CBCT was also used confirm the patency of the ostium and the osteomeatal complex.

### 2.2. Surgical Procedures and Flap Design

**Figure 1:**
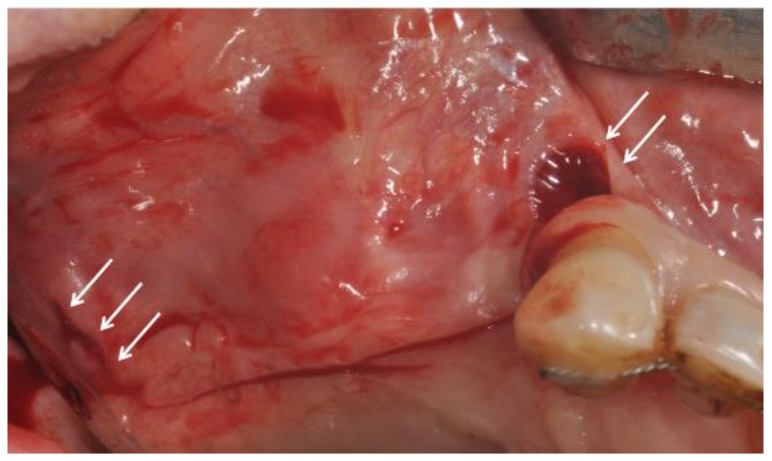
The trapezoidal flap with a relieving incision in the mesial and distal regions (arrows).

**Figure 2:**
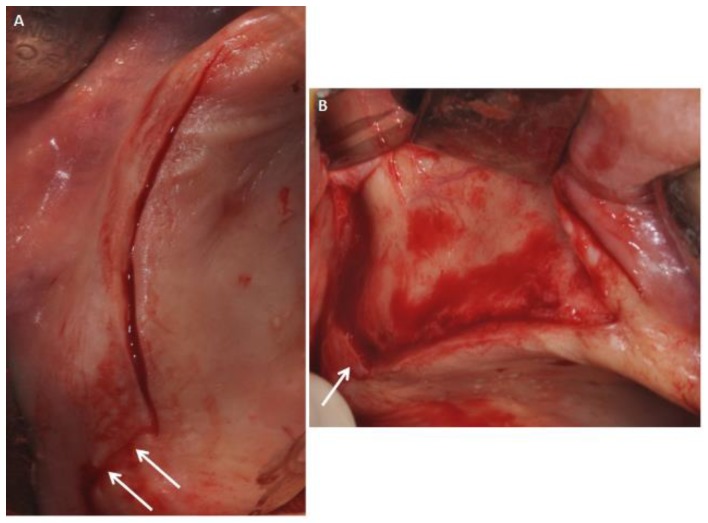
(**A**) The modified triangular flap with a relieving incision in the distal region without a mesial relieving incision (arrows). Flap in the case of an edentulous patient. (**B**) Flap elevated without difficulty.

**Figure 3:**
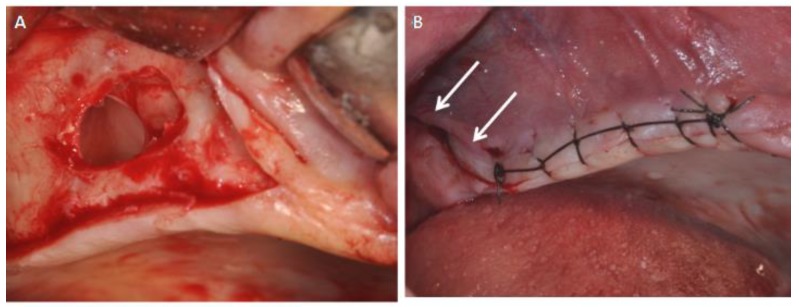
(**A**) Antrostomy access after dissection and elevation of the sinus membrane. (**B**) Flaps were carefully sutured while the distal relieving incision was left without suture as passive drains (arrows).

**Figure 4:**
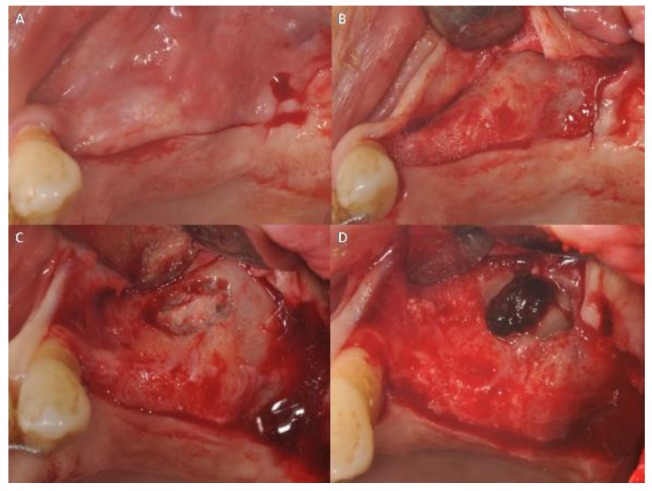
(**A**) Modified triangular technique. Flap in case of a partially edentulous patient. (**B**) The incision was continued by a sulcular incision starting near the mesialbuccal edge of the teeth. (**C**) The flap was elevated and the maxillary sinus lateral wall was exposed. (**D**) A bone window was created.

All patients were treated in the Outpatient Department of Oral Implantology, Center for Advanced Studies, Dental Research Division, UNINGÁ—Cachoeiro de Itapemirim, Brazil, and 39 implants were placed after six months of healing. All patients were evaluated for 14 days. The sites were randomly assigned to the test or control group by a computer-generated table, which was prepared using a balanced, randomly permuted sinus approach. We used free random sampling and random assignment application (Research Randomizer Version 4.0, Geoffrey C. Urbaniak and Scott Plous). All sinus lifts were performed by a single operator, experienced in performing both the trapezoidal (TZ) flap (Figure 1) and the modified triangular (MT) flap without anterior release (Figure 2A,B). The primary predictor variable was the flap type. Two different flap designs were used: the trapezoidal flap and the modified triangular flap. The trapezoidal flap technique was used for Group I: the incision was made on the top of the alveolar ridge horizontally with a relieving incision in the mesial and distal regions. The full-thickness flap was elevated with a flap elevator that had to be adherent to the bone so that the periosteum remained undamaged (Figure 1). The modified triangular flap type was used for Group II: the first part of the incision was similar to that in Group I ,although it extended mesially to the maxillary canine region if the patient was edentulous (Figure 3A,B). In the presence of teeth the incision was continued using a sulcular incision starting near the mesio-buccal edge of the teeth and then extended up to the midpoint of the buccal sulcus of the distal teeth (Figure 4A,B), with cutting of the interdental papilla (Figure 4C,D). 

Postoperative pain was scored by means of a 100-mm visual analogue scale (VAS) from 0 (no pain) to 100 (worst pain imaginable) at 2, 4 and 6 days in the first week post-surgery and then at 14 days. Pain intensity was classified into four categories. Swelling was recorded by verbal rating scale (VRS). In this scale, swelling was classified into four categories: a score of 1 referred to the absence of swelling, patients with an intra-oral swelling in the surgical zone scored 2, any extra-oral swelling in the surgical zone scored 3, and intense extra-oral swelling extending beyond the surgical zone scored 4 [31]. At each control the facial temperature was also measured. An independent examiner assessed the degree of swelling once daily, at the same time, for 14 postoperative days. 

Prior to surgery, patients rinsed with a chlorhexidine digluconate solution 0.2% for 2 min. Local anesthesia was obtained with Articaine^®^ (Ubistesin 4%—Espe Dental AG, Seefeld, Germany) associated with epinephrine 1:100,000. Full-thickness flaps were elevated to expose the alveolar crest and the lateral wall of the maxillary sinus. Using a round bur under cold (4–5 °C) sterile saline irrigation, a trap door was made in the lateral sinus wall. The door was rotated inward and upward with a top hinge to a horizontal position. The sinus membrane was elevated with curettes of different shapes, until it became completely detached from the lateral, inferior, and medial walls of the sinus. The maxillary sinus was filled with sterilized bovine bone particles mixed with venous blood (Re-Bone, Ubgen, Padova, Italy). No perforation of the sinus membrane was evident in 25 of the cases. A small perforation was evident in five cases, two in group I and three in group II. All small perforations appearing in the sinus membrane were repaired with autologous platelet gel [32]. Flaps were carefully sutured with Polimid 4.0 (Sweden & Martina, Due Carrare, Italy) in Group I, while in Group II the distal relieving incision was left without suture as passive drains while the flap was sutured. Amoxicillin (1 g, 2 times per day) was prescribed for one week. Patients allergic to penicillin were prescribed clindamycin 300 mg twice a day for 6 days. The therapy was standardized, and the dose of anti-inflammatory was homogeneous for all patients. To help reduce symptoms, analgesic medication (ibuprofen 600 mg) 2 h following surgery and every 6 h afterward was prescribed, to be continued for 3 days following surgery in all patients. This ensured that the medication acted prior to the local anesthetic wearing off, when it could be more difficult to control the pain. To help drainage from maxillary sinus ostium and to reduce or eliminate the feeling of a stuffy nose, the nasal decongestant Mometasone furoate monohydrate (Rinelon spray, Msd Italia Srl, Rome, Italy) was also prescribed 1 day before surgery and every 12 h following surgery, to be continued for 6 days following surgery. No corticosteroids were prescribed. To reduce any side effects (nausea or upset stomach), all the patients were encouraged to take the medications with a large glass of fruit juice.

Sutures were removed two weeks after surgery. Follow-up surgical visits were scheduled at 2, 4 and 6 days in the first week and at 14 days to check the course of healing. The recall program included assessments of VAS, VRS, and facial temperature assessment.

### 2.3. Temperature Measurements

Thermal measurements were performed in a climate-controlled room (temperature: 22–24 °C, relative humidity: 50 ± 5%, with no direct ventilation on the face of patients).

Facial temperature of the operated side was obtained using a 14-bit digital infrared camera (FLIR SC660 QWIP, Flir Systems, Danderyd, Sweden). The acquisition parameters were: 320 × 240 pixels focal plane array; 8–9 µm spectral range; 0.02 K noise equivalent temperature differences (NETDs); 50-Hz sampling rate; optics: germanium lens; f 20; and f/1.5. The camera was set 0.50 m away from the face for maximum spatial resolution. Images were acquired at a rate of 25 per second and subsequently re-aligned using an edge-detection based method implemented with in-house software. Temperature changes in the face were computed on the realigned thermal images. Thermographic data analysis was performed using FLIR QuickReport v.1.2 (FLIR Systems Inc., North Billerica, MA, USA), which includes a tool to obtain maximum, minimum, and average temperature of a user-defined area. The facial areas of interest were the malar and cheek areas.

### 2.4. Statistical Evaluation

A power analysis was performed using clinical software for determining the number of sinus needed to achieve statistical significance for quantitative analyses of VAS, VRS, and facial temperature. A calculation model was adopted for dichotomous variables (yes/no effect) by using the effect incidence designed to discern the reasons (85% for the TZ flap and 15% for the MT flap), with alpha = 0.05 and power = 80%.

The optimal number of samples for analysis was 14 sinus augmentations.

The correlation between temperature and pain at each time was assessed by a linear regression model; to assess the correlation of measurements throughout time, mixed effect repeated measures were performed. To evaluate the differences in the VAS, VRS, and facial temperature between the groups, one-way ANOVA statistical analysis was used. A value of 𝑝 ≤ 0.05 was considered to be statistically significant. Data treatment and statistical analysis were done by Excel (Microsoft Excel, Redmond, WA, USA) Origin (OriginLab, Northampton, MA, USA) and SPSS software (IBM, Armonk, NY, USA).

### 2.5. Trial Registration 

The trial is registered on the Brazilian Clinical Trials Registry/Registro Brasileiro de Ensaios Clínicos (ReBEC) (UTN: #U11111-1202-3138).

## 3. Results

### 3.1. Group I: TZ Flap

**Figure 5:**
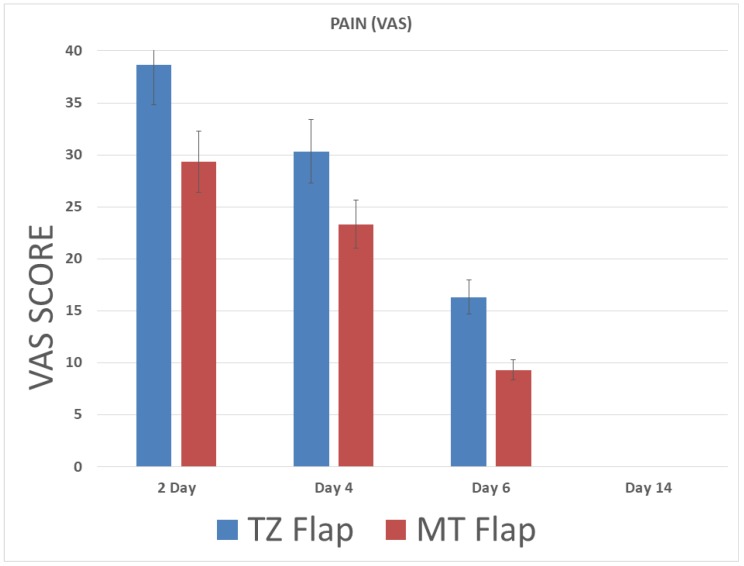
Pain measurements based on the VAS scores.

**Figure 6:**
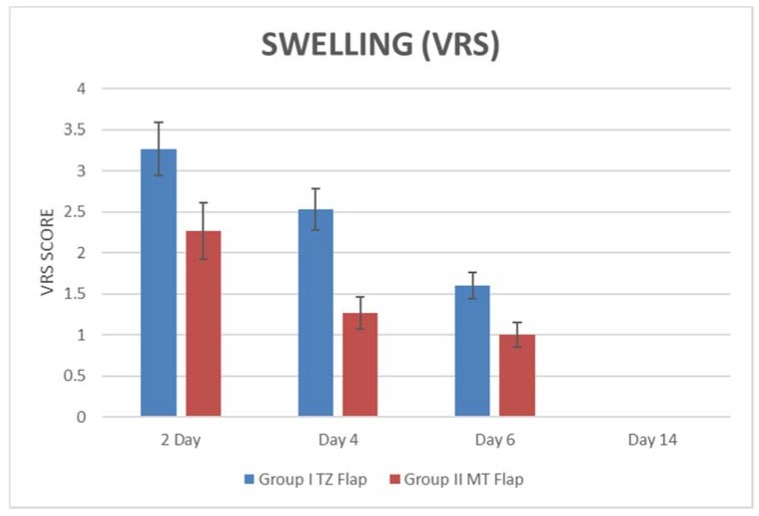
Swelling measurements based on the verbal rating scale.

**Figure 7:**
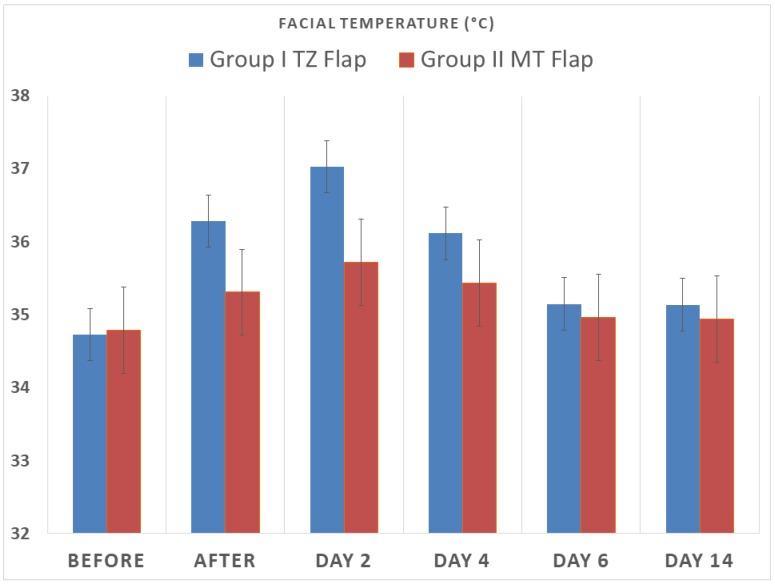
Facial temperature measurement before the procedure and after sinus augmentation.

**Figure 8:**
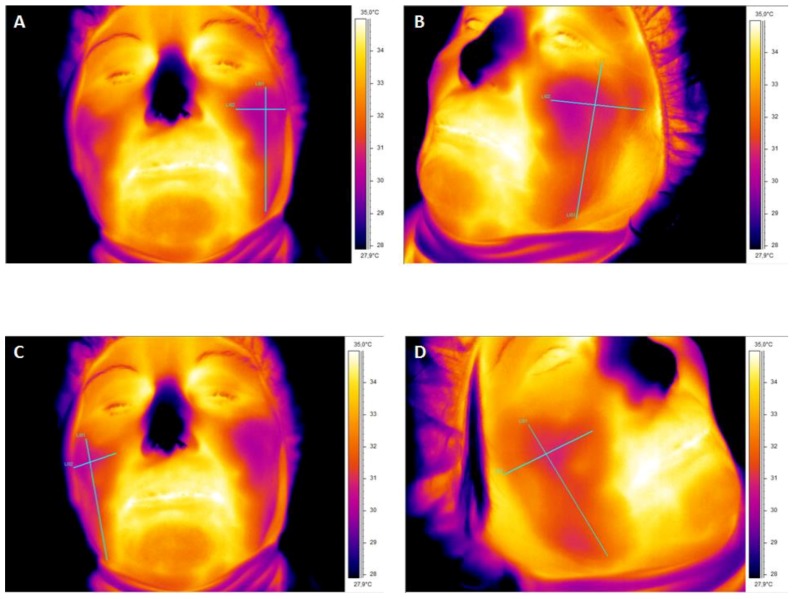
Thermographic scan of face after 2 days. (**A**,**B**) Trapezoidal flap. Measurements were taken ipsilateral to the side of sinus lifting. (**C**,**D**) Modified triangular technique. The surface temperature was lower than that using the trapezoidal flap.

All augmented sinuses healed well with no occurrence of symptoms indicating possible maxillary sinusitis. Based on the VAS scores, peak pain intensity was recorded at 2 days after surgery with a mean score of 38.67 ± 6.4 mm (Table 1 and Figure 5, Figure 6, Figure 7 and Figure 8A,B). After the second day, the pain intensity reduced significantly during the consecutive time intervals. The swelling score was 3.27 ± 0.59 on the second day and was reduced significantly after four days. There was no swelling on day 6 after the surgery (Table 1). Table 1 and Figure 5 and Figure 6 demonstrate the values of swelling and pain.

The difference in temperature before the procedure (basal measurement) and immediately after sinus augmentation was 1.55 °C (Table 1 and Figure 7). The difference in temperature increased on day 2 (Figure 8A,B) and reduced significantly on day 6 (Table 2).

### 3.2. Group II: MT Flap

No postoperative paresthesia or signs of infection were seen during the entire healing period.

All augmented sinuses healed well with no occurrence of symptoms indicating possible maxillary sinusitis. The clinical healing of the surgical area was uneventful in all patients. Wound dehiscence or infection were not observed in any of the cases. The VAS pain scores were mild on the second and fourth day and were lower than in Group I. The pain intensity was recorded at 2 days after surgery with a score of 29.33 ± 7.03 mm, which is mild (Table 1 and Table 2). At four days the pain intensity reduced significantly, with a score of 23.33 ± 6.17. Swelling scores were 2.27 ± 0.45 on the second day, 1.26 ± 0.45 on the fourth day, and 1 ± 0 the sixth day; day 14 the score was 0 (Table 1). Table 1 shows the values of swelling and pain. The swelling scores at all times were lower than in Group I. The difference in temperature before the procedure (measurement basal) and immediately after sinus augmentation was 0.51 °C (Table 2). After surgery the difference in temperature reduced significantly on days 4 and 6, and on day 14 no difference was registered (Table 1 and Figure 7).

The difference in temperature increased on day 2 (Table 2 and Figure 8C,D), and reduced significantly on day 4 (Table 2 and Figure 7).

### 3.3. Statistical Evaluation

Significant differences in VAS scores were found between the two groups at 2 days (*p* = 0.0004), 4 days (*p* = 0.002), and 6 days (*p* = 0.01); no differences were found at 14 days.

Significant differences in VRS scores were found between the two groups at 2 days (*p* = 0.000018), 4 days (*p* = 0.0008), and 6 days (*p* = 0.009); no differences were found at 14 days.

A significant difference in temperature was found between the two groups before and after surgery (*p* = 0.00013), at 2 days (*p* = 0.003) and at 4 days (*p* = 0.02); no differences were found at 6 days (*p* = 0.18) and at 14 days.

## 4. Discussion

Different biomaterials are currently used for sinus lifting with the lateral approach [33] and the features of a bone substitute are critical factors for the success of bone augmentation [34].

Sinus lift with a lateral approach is a painful and uncomfortable treatment, causing postoperative pain and swelling. The results of this clinical study show the significant effectiveness of the modified triangular flap for performing lateral sinus lifts, with a reduction in postoperative pain and swelling. The results of this study show statistically significant differences for pain, swelling, and temperature between two types of flap under observation.

This is the first study on the utilization of the modified triangular flap in sinus lift procedures. Unfortunately, most oral surgeries are accompanied by some degree of discomfort, with pain and swelling. Postoperative pain evaluation in patients can be a difficult task, since it is a multidimensional experience that includes sensory and affective components that are often hard to discriminate with the existing scores [35,36]. For this reason, a complementary observational pain measurement should be used to assess pain intensity.

The most common way to assess postoperative pain in the reviewed studies has been the use of visual analog scales (VAS) and calculation of the amounts of analgesics consumed [37]. Swelling can be quantified in different ways. In the case of removal of impacted third molars, one of the most commonly used methods is that of Amin and Laskin [38], which uses suture thread grasped with two mosquito forceps to measure distances at the following reference points: from the external palpebral angle to the gonial angle on the operated side; from the lower margin of the tragus to the external angle of the oral commissure; and from the lower margin of the tragus to the midpoint of the chin symphysis. These assessments are difficult to apply for the evaluation of pain and swelling in sinus surgery.

To overcome these difficulties, we used infrared thermography. This has been used for the evaluation of temperature during implant preparation [39] and the treatment of diseases such as neuropathic pain, postherpetic neuralgia, whiplash injuries, inflammatory arthritis, temporomandibular joint disorders, headache, and myofascial pain syndromes [40]. It is also used as a diagnostic guide and therapeutic tool [41], in different anatomical locations [42]. Several factors influence postoperative pain and swelling. Age, sex, duration of surgery, and other environmental factors potentially influence the discomfort experienced after implant surgeries. However, the duration of surgery influences post-operative pain. Patients with longer surgical times experience more pain post-operatively [43].

In the present study, the two control and test groups were uniform with respect to given factors; the patients were chosen in the same age range, the groups had approximately the same number of men and woman, and the durations of the surgeries were all between 20 to 25 min. All the patients were prescribed similar pharmacological therapy. In fact, skin temperature is the most important method to determine the presence of tissue swelling and inflammation. 

The flap design is important in oral surgery. In fact, the ultimate goal in sinus surgery is not only the bone augmentation but also the preservation of periodontal tissue, use of suitable surgical techniques, and the reduction of postoperative swelling. Usually, in sinus surgery with a lateral approach, full-thickness mucoperiosteal flap elevation with a crestal and bi-lateral oblique releasing mesial and distal incision is used. However, this could also interfere with the blood supply from the vestibule and might inhibit adequate blood supply to the flap. The vascularization of the maxilla and buccal vestibule maxilla is densely vascularized by two vessels: the posterior superior alveolar artery and the infraorbital artery. The infraorbital artery reaches the face through the infraorbital foramen and supplies the lower eyelid, cheek, and lateral nose. It anastomoses with branches of the transverse facial, ophthalmic, buccal, and facial arteries [44]. The maxillary artery has been shown to have enormous individual variations in its topography, diameter, and size. Well known is the anastomoses between posterior superior alveolar artery and infraorbital artery that supply the lateral sinus wall and overlying membrane. The local oral mucosa as well as the mucous membrane of the lateral maxillary sinus are vascularized by these two vessels in a double arterial circle [45]. Based on the arterial blood supply, it would be best to avoid vertical mesial release, because in this area the branches of the infraorbital artery are found. 

The flap should be designed to minimize disturbance of the blood supply, and the surgical site needs to be securely covered [46]. Ideally, a mid-crestal or a slightly palatal incision should be performed, leaving at least 3 mm of attached tissue on the facial aspect of the incision, with anterior and posterior vertical release. These vertical incisions should be at least 5 mm away from the planned osteotomy (Figure 1). A full-thickness mucoperiosteal flap is then raised and the lateral aspect of the maxillary sinus is exposed. The infraorbital foramen should be avoided during mesial release; precautions should be taken not to injure the neurovascular bundle during the preparation of the door and retraction of the flap. In the case of the modified triangular flap, risk of injuring the infraorbital neurovascular does not exist.

## 5. Conclusions

The results of this clinical study show significant effectiveness of the modified triangular flap in the sinus lift procedure for reducing pain and swelling. It is probable that the posterior passive drains reduce swelling and pain when the modified triangular flap is used. In fact, the distal relieving incision was left without sutures. This passive drain promotes the spontaneous leaking of the inflammatory exudate and reduces postoperative swelling and pain. The MT flap cannot damage the lateral blood supply, which runs in a posterior to anterior direction [47]. In particular, the anterior vertical incision may have a reduced blood supply. The reduced pain and swelling found in MT flaps are due to the spontaneous passive drains and to a greater blood supply. Based on the outcome of the present study, the MT flap is effective in reducing pain and swelling after sinus lifting.

In conclusion, a modified triangular flap can be used with success in sinus augmentation procedures and is preferable to the trapezoidal flap.

## Figures and Tables

**Table 1:** Pain and swelling score recording at experimental times. At 14 days there was no symptoms detected in patents of both study groups.

	PAIN (VAS)				SWELLING (VRS)			
Group I TZ Flap	Day 2	Day 4	Day 6	Day 14	Day 2	Day 4	Day 6	Day 14
Min	30	15	10	0	2	2	1	0
Max	50	50	30	0	4	4	3	0
Mean	38.67	30.33	16.33	0	3.27	2.53	1.60	0
SD	6.40	9.34	6.11	0	0.59	0.74	0.82	0
Group II MT Flap	Day 2	Day 4	Day 6	Day 14	Day 2	Day 4	Day 6	Day 14
Min	20	10	0	0	2	1	0	0
Max	40	30	20	0	3	2	1	0
Mean	29.33	23.33	9.33	0	2.27	1.26	1	0
SD	7.03	6.17	7.98	0	0.45	0.45	0	0

VRS: verbal rating scale; VAS: visual analogue scale; TZ: trapezoidal; MT: modified triangular.

**Table 2:** Facial patients temperature values of both study groups. Infrared thermal detections were performed at experimental times.

	Temperature (°C)					
Group I TZ Flap	Before	After	Day 2	Day 4	Day 6	Day 14
Min	34.20	35.2	35.60	34.9	34.50	34.3
Max	35.30	36.9	37.80	36.9	36.30	35.7
Mean	34.73	36.28	37.03	36.11	35.15	35.13
SD	0.37	0.44	0.53	0.55	0.55	0.52
Group II MT Flap	Before	After	Day 2	Day 4	Day 6	Day 14
Min	34.20	34.2	34.10	34	34.20	34.3
Max	35.60	36.8	38.10	36.8	36.30	35.7
Mean	34.79	35.30	35.72	35.43	34.96	34.94
SD	0.44	0.75	1.11	0.92	0.59	0.50
